# Comprehensive characterization of B7 family members in NSCLC and identification of its regulatory network

**DOI:** 10.1038/s41598-022-26776-w

**Published:** 2023-03-15

**Authors:** Mintao Xiao, Chunrong Pang, Shixin Xiang, Yueshui Zhao, Xu Wu, Mingxing Li, Fukuan Du, Yu Chen, Fang Wang, Qinglian Wen, Zhangang Xiao, Zhongming Yang, Jing Shen

**Affiliations:** 1 Laboratory of Molecular Pharmacology, Department of Pharmacology, School of Pharmacy, Southwest Medical University, Luzhou, 646000 Sichuan People’s Republic of China; 2 South Sichuan Institute of Translational Medicine, Luzhou, Sichuan People’s Republic of China; 3 Sichuan Clinical Research Center for Birth Defects, Luzhou, Sichuan People’s Republic of China; 4 Department of Obstetrics, Affiliated Hospital of Southwest Medical University, Luzhou, People’s Republic of China; 5 Department of Oncology, Affiliated Hospital of Southwest Medical University, Luzhou, Sichuan People’s Republic of China; 6 Department of Oncology and Hematology, Hospital (T.C.M) Affiliated to Southwest Medical University, Luzhou, Sichuan People’s Republic of China; 7 Department of Pharmacy, University-Town Hospital of Chongqing Medical University, Chongqing, People’s Republic of China

**Keywords:** Cancer, Computational biology and bioinformatics, Immunology, Molecular biology

## Abstract

B7 family members act as co-stimulatory or co-inhibitory molecules in the adaptive immune system. Thisstudy aimed to investigate the dysregulation, prognostic value and regulatory network of B7 family members in non-small cell lung cancer (NSCLC). Data for lung adenocarcinoma (LUAD) and lung squamous cell carcinoma (LUSC) patients were extracted from public databases. Patient prognosis was determined by Kaplan–Meier analysis. The downstream signaling pathways of B7 family were identified via GO and KEGG analysis. The key B7 related genes were selected by network, correlation and functional annotation analysis. Most B7 family members were dysregulated in LUAD and LUSC. The expression of B7-1/2/H3 and B7-H5 were significantly associated with overall survival in LUAD and LUSC, respectively. The major pathway affected by B7 family was the EGFR tyrosine kinase inhibitor resistance and ErbB signaling pathway. MAPK1, MAPK3 and MAP2K1 were pivotal B7 related genes in both LUAD and LUSC. This study reveals an overall dysregulation of B7 family members in NSCLC and highlights the potential of combination use of tyrosine kinase inhibitors or MEK/ERK inhibitors with B7 member blockade for NSCLC treatment.

## Introduction

Lung cancer is the most common cancer type with 2.1 million new cases diagnosed in 2018, accounting for 12% of newly diagnosed cancer cases^1^. It is also the first cause of worldwide cancer death with estimated 1.6 million deaths annually that accounted for 20% of total cancer-related death with very low 5-year survival rate^1–3^. According to the IARC report on cancer incidence and mortality in 2020: 2,206,771 new cases (about 11.4% of all cancer cases) and 1,796,144 new deaths of lung cancer (about 18.0% of cancer deaths) in 2020. Lung cancer is the leading cause of cancer death and is also the most common type of cancer in men and the second most common type of cancer in women^4^. Non-small cell lung cancer (NSCLC) accounts for approximately 85% of lung cancer^5^. Lung adenocarcinoma (LUAD) and lung squamous cell carcinoma (LUSC) are the two most common subtypes of NSCLC, accounting for 65–70% of all lung cancers^6^. Smoking is currently considered to be the most important risk factor for lung cancer^7–9^. A majority of patients are diagnosed with advanced or metastatic disease without apparent symptoms^10,11^. Currently, there are limited treatment methods for NSCLC, with chemotherapy being the main clinical option^2^ In recent years, tumor immunotherapy has been applied in some cases in NSCLC patients and the advancement of it has improved patient survival and reduced suffering in some cancer patients, providing important information for diagnosis and treatment of NSCLC^6,12^.

The B7 family is defined as co-stimulating or co-inhibiting molecules in the activation of T cells and their ligands and receptors play critical roles in the adaptive immune response and malignant tumors^13^. It belongs to IgSF (immunoglobulin super-family) and its outer membrane domain contains a different amount of immunoglobulin domain^14^. In the past decades, the B7 family has been found to include 10 members: B7-1(CD80), B7-2(CD86), B7-DC(PD-L2 or CD273), B7-H1(PD-L1 or CD274), B7-H2(ICOSLG), B7-H3(CD276), B7-H4(VTCN1), B7-H5(VISTA), B7-H6(NCR3LG1) and B7-H7(HHLA2)^15^. Studies have shown that many inhibitory B7 family members are highly expressed in tumors and participate in tumor cell immunosuppression through a negative second signal^16–18^. Abundant inhibitory signals may weaken the response of T cells^19^. Experimental evidence suggests that manipulation of the B7 family may affect anti-tumor immunity, thus the B7 family and its receptors hold great potential as targets for tumor immune checkpoint therapy^20^.

To date, the pathogenesis of NSCLC, including the epigenetic regulation of mRNA and protein changes has not yet been fully elucidated, and the molecular mechanism needs to be further explored to make certain about the progress of therapeutic options for the occurrence and development of NSCLC. Strikingly, notwithstanding these open questions, increasing studies have suggested that the introduction of targeted therapy and immune checkpoint inhibitors has greatly improved the treatment of patients with NSCLC compared with traditional therapeutic approaches^8,9^. Some genes also contribute to dissimilar degrees of cell invasion in NSCLC^21^. Therefore, formulating related agents for certain immune checkpoint inhibitors to effectively inhibit the occurrence and development of NSCLC cells has become another mainstream way^22^. In this study, based on the public data, we analyzed the mRNA expression level, gene alterations, DNA methylation, prognostic value, functional network of the B7 family and identified the B7 family regulatory network in NSCLC. The findings may help better understand the molecular mechanism of NSCLC and provide information on favorable combined immunotherapy of NSCLC patients in the future.

## Results

### Dysregulation of B7 family members in NSCLC

**Figure 1:**
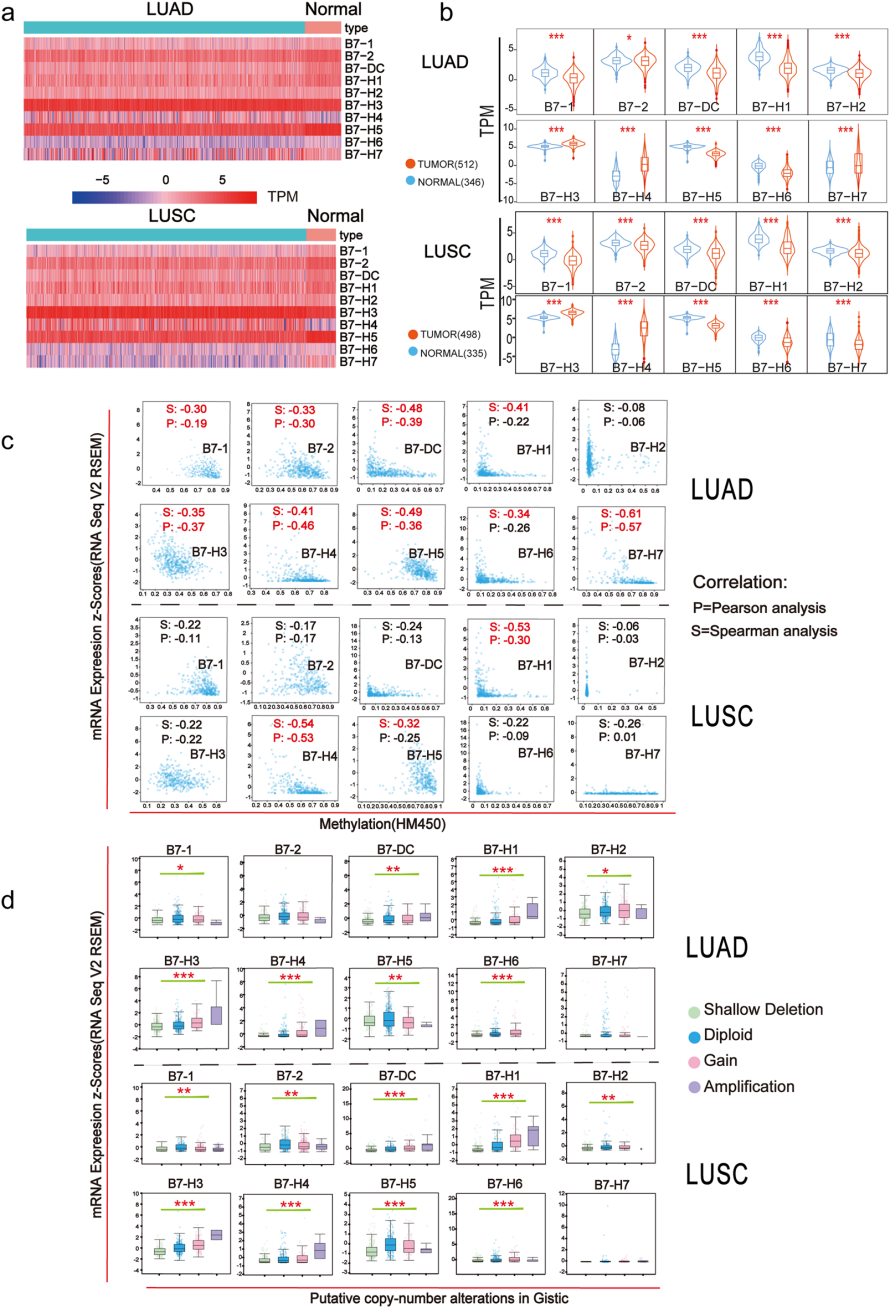
Altered expression and underlying mechanism of B7 family in different subtypes of NSCLC. (**a**) The expression profile of B7 family was displayed in heatmap in normal and tumor tissues of different NSCLC patients with data extracted from TCGA database. (**b**) The violin plots showed the average expression level of B7 family members in normal and tumor tissues based on TCGA and GTEX data. Expression level is mapped by Transcripts Per Kilobase of exon model per Million mapped reads (TPM). (LUAD:512 tumor samples and 347 normal samples; LUSC:498 tumor samples and 336 normal samples). (**c**) Correlation between promoter DNA methylation and the expression of B7 family. (**d**) The relationship between expression and copy numberstatus of B7 family members (*P < 0.05; **P < 0.01; ***P < 0.001).

Heatmap showing the expression levels of B7 family member in LUAD and LUSC was constructed using TCGA data (Fig. 1a). Overall, the expression levels of B7-H3 and B7-H5 were relatively higher while the expression level of B7-H6 was lower than that of other B7 members in both LUAD and LUSC. TCGA and GTEx data were then combined to compare the expression levels of B7 family member between normal and tumor tissues in LUAD and LUSC (Fig. 1b). The results showed that B7-1, B7-2, B7-DC, B7-H1, B7-H2, B7-H5, B7-H6 were consistently down-regulated in both LUAD and LUSC, but B7-H3 and B7-H4 were up-regulated. Particularly, the level of B7-H7 was increased in LUAD but decreased in LUSC. To unveil the dysregulation mechanism of B7 family members in NSCLC, we investigated the relationship between the expression of B7 family members and DNA methylation and copy number alteration, respectively (Fig. 1c, d). The mRNA expression of B7-1/2/DC/H3/H4/H5/H7 were inversely correlated with DNA methylation level in LUAD and the expression of B7-H1 and B7-H4 were also negatively associated with DNA methylation in LUSC (Fig. 1c), suggesting that the dysregulation of B7 members might be partially due to promotor DNA hyper/hypo-methylation in NSCLC. Moreover, the expression level of B7-1, B7-DC, B7-H1, B7-H2, B7-H3, B7-H4, B7-H5, B7-H6 were significantly associated with gene amplification, diploid and copy number increase in LUAD. In the meantime, the expression of B7-1, B7-2, B7-DC, B7-H1, B7-H2, B7-H3, B7-H4, B7-H5, B7-H6 were significantly associated with copy number status in LUSC (Fig. 1d). Collectively, our data suggests that both DNA methylation and copy number alteration may be involved in the dysregulation of B7 family members in NSCLC.

### Genetic alterations of B7 family members in NSCLC

**Figure 2:**
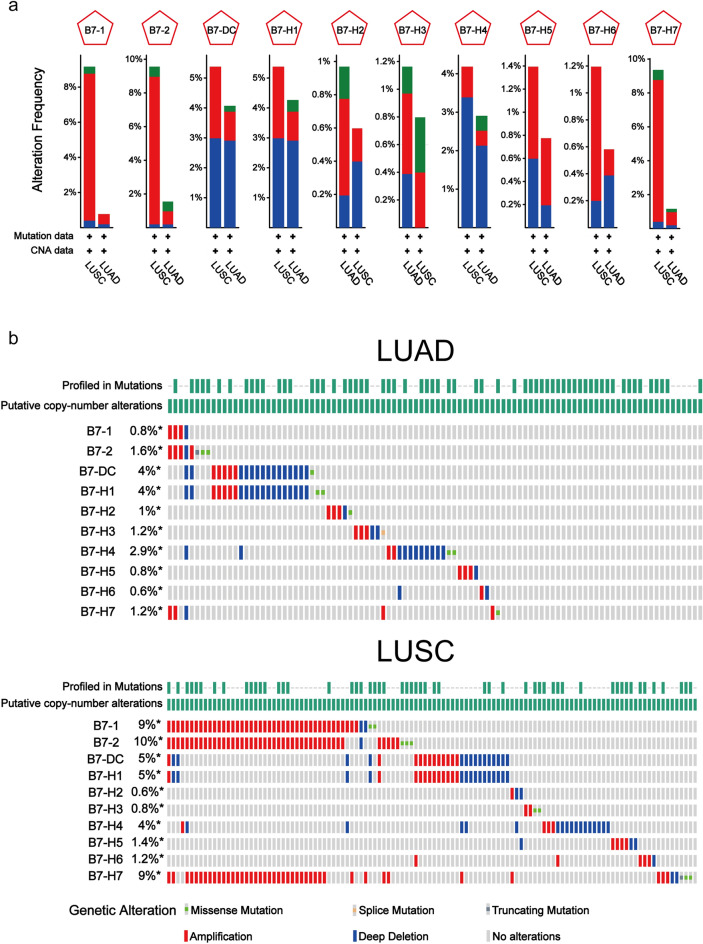
Genetic alterations of B7 family in different subtypes of NSCLC. (**a**) The frequency of genetic alterations in B7 family members in NSCLC. The types of changes include mutations (green), amplifications (red), and deep deletions (blue). (**b**) The gene mutation rate of B7 family members was analyzed, including missense mutations (green), truncation mutations (dark gray), amplification (red), deep deletion (blue) and no change (gray).

To further understand the dysregulation of B7 members in NSCLC, we also determined their mutations. The frequency of B7 member mutation, amplification and deep deletion in LUAD and LUSC was shown in Fig. 2a. Overall, the mutation rates of B7 members were relatively higher in LUSC than LUAD. B7-1, B7-2 and B7-H7 exhibited higher mutation rates than other B7 members in LUSC and the major mutation type for them and B7-H5, B7-H6 was amplification. The major mutation type found for B7-DC, B7-H1 and B7-H4 was deep deletion. At the same time, Fig. 2b demonstrated the total mutation rate and different mutation types of B7 members. Similar with the results in Fig. 2a, the mutation rates for B7-DC, B7-H1 and B7-H4 were relatively higher in both LUAD (4%, 4% and 2.9%) and LUSC (5%, 5% and 4%). But the mutation rates were even higher for B7-1 (9%), B7-2 (10%) and B7-H7 (9%) in LUSC but not LUAD.

### Association of B7 family member expression with clinicopathological parameters and survival

**Figure 3:**
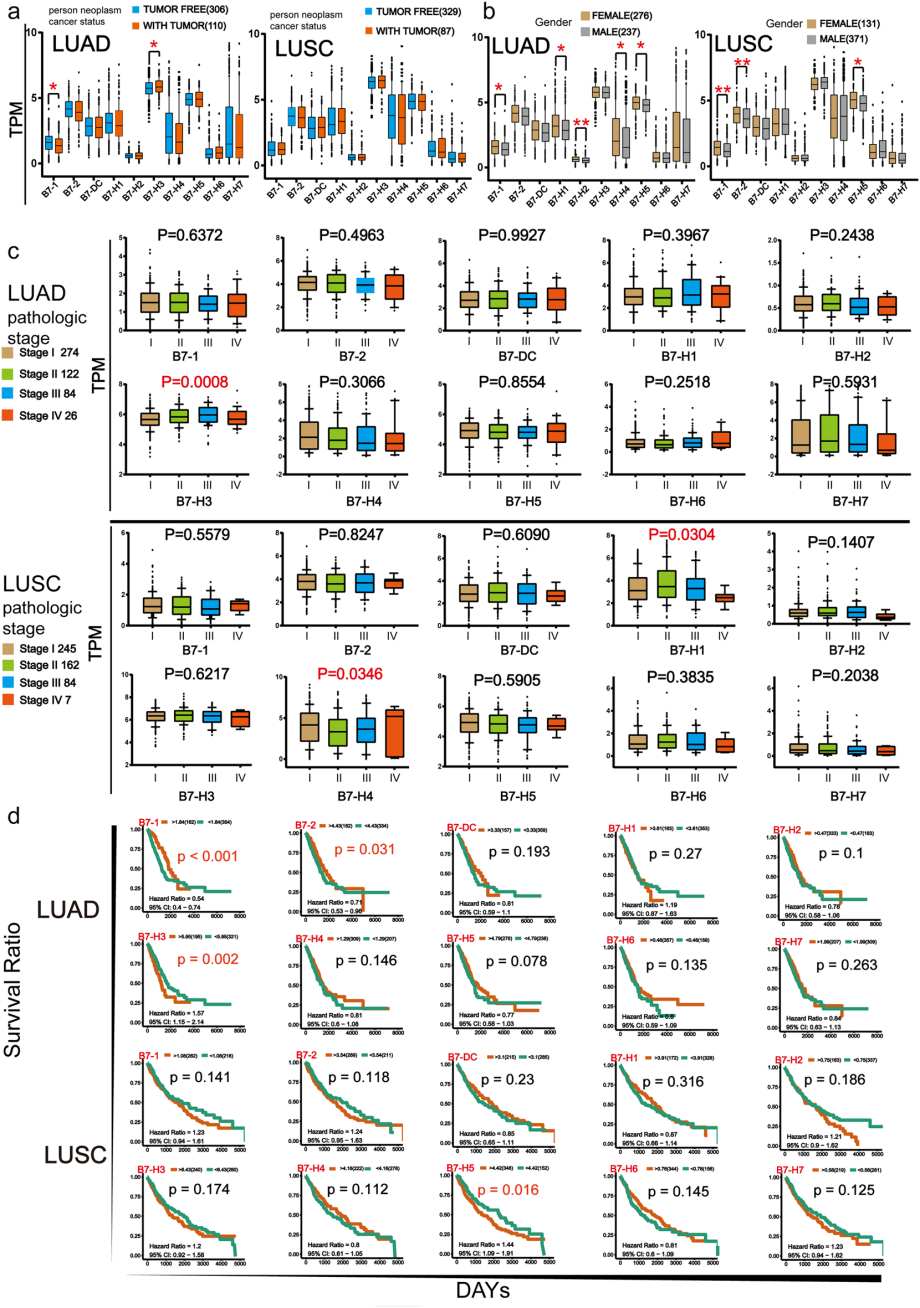
The association ofB7 family expression with clinicopathological parametersand patient survival. The relationship between the expression of B7 family members and (**a**) cancer status (*P < 0.05; **P < 0.01 and ***P < 0.001), (**b**) gender and (**c**) pathologic stages (Stage I, Stage II, Stage III, Stage IV). (**d**) Kaplan–Meier survival curves for different subtypes of NSCLC patients stratified according to high vs low expression of B7 family.

We further assessed the association between B7 family member expression and some clinicopathological parameters including cancer status (with tumor, tumor free), gender (male, female) and pathological stage (stage I, stage II, stage III, stage IV) (Fig. 3a–c). The expression level of B7-1 was significantly lower while the expression level for B7-H3 was higher in patients with tumor after treatment than in patients who are tumor free in LUAD (Fig. 3a). Moreover, the expression of B7-1/H1/H2/H4/H5 was significantly lower in male than in female in LUAD. Meanwhile, the expression of B7-1/2/H5 was significantly lower in male than in female in LUSC (Fig. 3b). For pathological stage, the expression level of B7-H3 exhibit significant difference in LUAD increasing from stage I to stage III but decreasing in stage IV. Compared with stage I, the expression of B7-H1 was decreased and B7-H4 was increased in stage IV in LUSC (Fig. 3c).

Subsequently, we also explored the relationship between expression of each B7 family member with overall survival (OS) (Fig. 3d). According to Kaplan–Meier analysis, the expression of B7-1/2/H3 was associated with patient survival in LUAD and the expression of B7-H5 was associated with patient survival in LUSC. In LUAD, higher expression of B7-1 and B7-2 was associated with prolonged OS while elevated expression of B7-H3 was associated with worse OS. In LUSC, higher expression of B7-H5 predicted shortened OS. Consistent with our finding, high expression level of B7-H3 and B7-H5 has been reported to be associated with poor OS in NSCLC^23,24^.

### Function and pathway analysis for differentially expressed proteins

**Figure 4:**
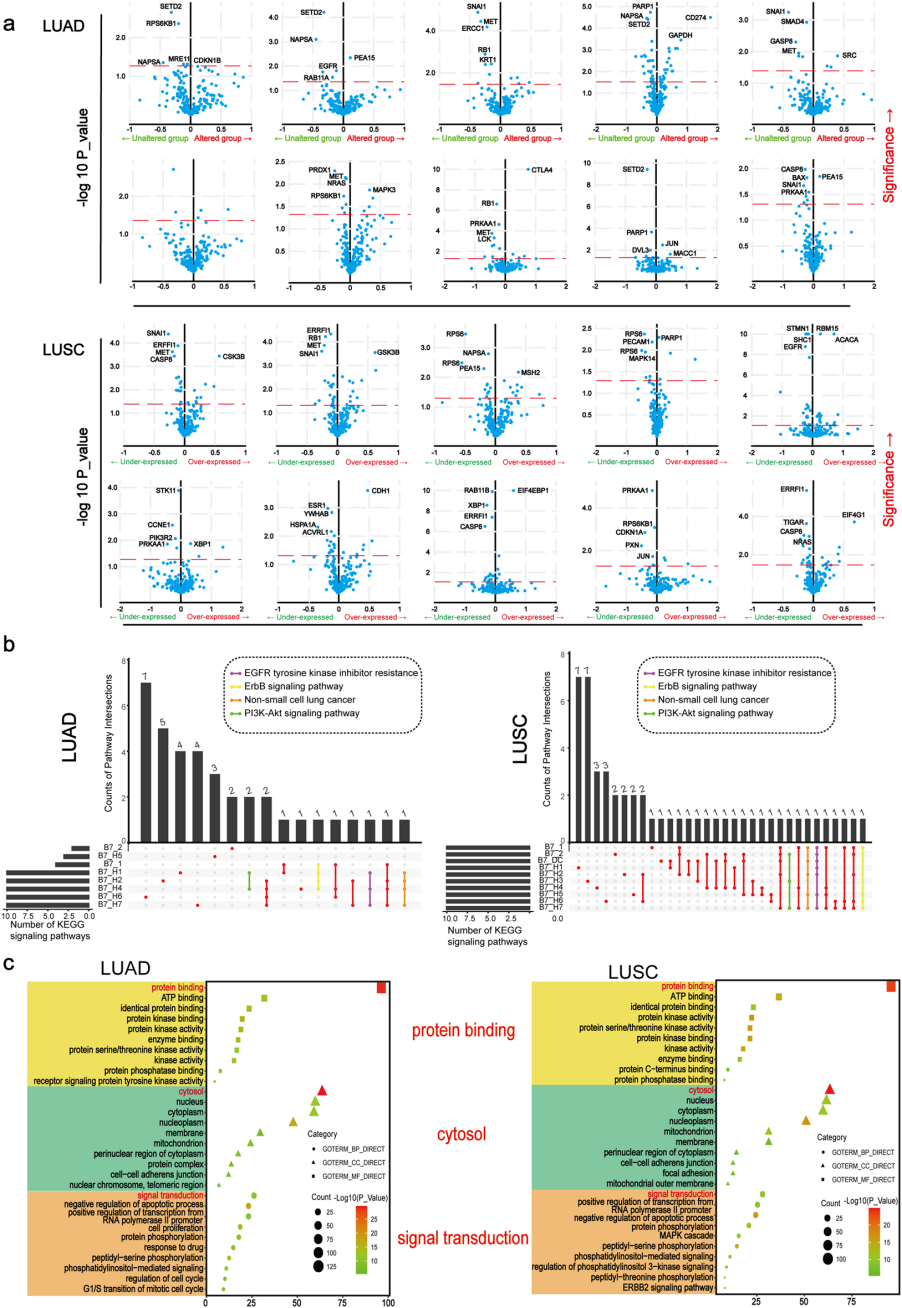
GO and KEGG enrichment analysis of B7 family. (**a**) Enrichmentof differentially expressed proteins of B7 family in NSCLC. − log10 (*P* value) = 1.30 is considered to be cut-off value for difference. The distance of expression level change was based on logarithmic ratio (mean in altered/mean in unaltered). (**b**) Intersection of top10 important KEGG signaling pathways for each B7 family member. (**c**) The influence of B7 family on downstream processes were analyzed by GO enrichment analysis via DAVID.

Changes in protein expression caused by B7 family gene alterations were extracted from the Reverse Phase Protein Arrays (RPPA) database and analyzed in cBioportal. Significantly changed proteins in LUAD and LUSC were screened out in RPPA, respectively (Fig. 4a). The intersection of the top10 most significant signaling pathways enriched by different B7 family members was shown in Fig. 4b. The ErbB signaling pathway and EGFR tyrosine kinase inhibitor resistance were the most significant pathway influenced by B7 member alteration in both LUAD and LUSC (Fig. 4b). PI3K-Akt signaling pathway and Non-small cell lung cancer were also among the top pathways that the differentially expressed proteins were involved in for both NSCLC subtypes. Interestingly, these significant pathways all collectively presented in the ErbB signaling pathway (Supplementary Fig. 1)^25–27^. At the same time, the three major GO(Gene Ontology) terms for the differentially expressed proteins were also analyzed (Fig. 4c). The results for LUAD and LUSC were very similar, with protein binding being the major molecular function for the differentially expressed proteins. Signal transduction was the most important biological process and the most significant cellular component was cytosol.

### MAPK1/3 and MAP2K1 are important B7 family member related genes in NSCLC

**Figure 5:**
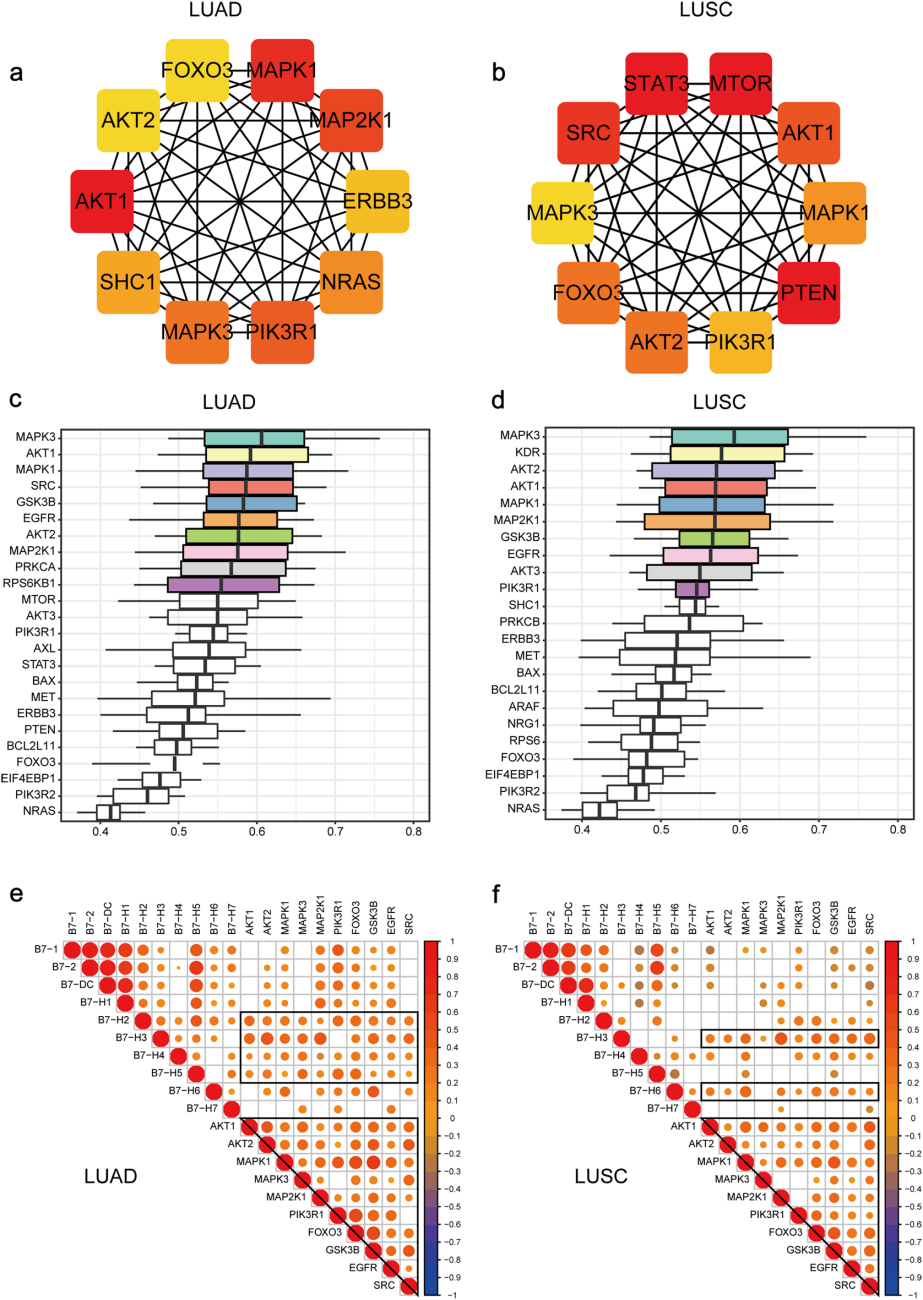
Identification of pivotal B7 family related genes in NSCLC. (**a**, **b**) Cytoscape identified top10 important differentially expressed proteins regulated by B7 family in both LUAD and LUSC.The intensity of color showed the importance of genes in the networks. (**c**, **d**) Similarity of differentially expressed proteins based on Gene ontology in different GO terms inLUAD and LUSC. (**e**, **f**) Correlation analysis of differentially expressed protein-coding genes enriched in the PI3K-Akt signaling pathway and MAPK signaling pathway in LUAD and LUSC. Here, Pearson correlation coefficient was used to quantify the correlation.

For a better understanding of the interactions amongst these differential proteins in the EGFR tyrosine kinase inhibitor resistance and ErbB signaling pathway and to identify the most important proteins, the functional network diagram of the top 10 important proteins were identified using Cytoscape(Fig. 5a, b). These crucial proteins displayed complex interactions in both PI3K-Akt signaling pathway and MAPK signaling pathway. At the same time, GOSemSim and org.hs.eg.db R package were used to calculate the similarities of the differentially expressed proteins in the EGFR tyrosine kinase inhibitor resistance and ErbB signaling pathway by considering the GO topology (Fig. 5c, d). Moreover, MAPK1/3 and MAP2K1 exhibited high functional similarities among these differentially expressed proteins. The expression levels of B7 family and these differentially expressed protein-coding genes were utilized for calculating the correlation (Fig. 5e, f). Overall, the cascade formed by the expression of genes related to the PI3K-Akt signaling pathway and MAPK signaling pathway also exhibited extremely high internal correlation. MAPK1/3 and MAP2K1 were found to be important differential proteins in both LUAD and LUSC and exhibited good correlation with B7 members. MAPK1/3 and MAP2K1 were positively and significantly correlated with B7-H2/H3/H4/H5 in LUAD and also positively and significantly correlated with B7-H3/H6 in LUSC. Intriguingly, MAPK1/3 and MAP2K1 were critical components of ErbB/MAPK signaling pathway as well (Fig S1a). All of this evidence suggests that the actions of the ErbB/MAPK signaling pathway and ErbB/PI3K-Akt signaling pathway would be affected by the regulation of the B7 family.

## Discussion

B7 family members act as the secondary co-stimulatory or co-inhibitory signals regulating T cell activation and play an important role in the immune response to NK cells^15^. In terms of the developing two-signal theory of T cells, the binding action of T cell receptor (TCR) alone does not activate T cells but signals from co-stimulating and co-suppressing molecules can regulate TCR signals^14^. For example, signals from the B7 family and its ligand CD28 family can bind to TCR to affect the strength and duration of the immune response, which not only plays adramatic role in the anti-tumor process but can also maintain its own tolerance and thus inhibits the autoimmunity^13,14,28^. Expressions of some B7 family members are associated with the development of a number of diseases, such as extramammary Paget disease (EMPD), diffuse large B-cell lymphoma (DLBCL), renal cancer and advanced gastric cancer, suggesting its importance in tumorigenicity and immune escape^15,29,30^. Due to the unclear expression pattern, regulation mechanism and downstream signal transduction pathway of B7 family in NSCLC, we herein provide a comprehensive study of B7 family using a variety of bioinformatics analysis.

In the present study, we first examined the expression profile of B7 family in NSCLC (Fig. 1a, b). There were consistently increased levels of B7-H3/H4 and decreased B7-1/2/DC/H1/H2/H5/H6 in the two NSCLC subtypes. B7-1 (CD80) and B7-2 (CD86) are co-stimulatory molecules which triggers T cell activation. Low expression of B7-1 and B7-2 has been found in different tumors and positivity of them are usually associated with better OS^31–33^, which is also found in our analysis. B7-H2 is a co-stimulatory member of the B7 family and interaction of B7-H2 and its receptor ICOS promotes T cell responses. However, the role of B7-H2 in cancer is complicated. On one hand, B7-H2 expressing myeloma cells induced CD4+ T cells to proliferate and produce soluble factors which stimulate myeloma cell proliferation^34^. On the other hand, ICOS/B7-H2 signal enhances secondary responses by CD8(+) T cells and improves effectiveness of cancer therapy^35,36^. Nonetheless, selective down-regulation of B7-H2 on tumor cells is deemed as a means of tumor immune escape^37,38^. B7-H3 and B7-H4 has been widely reported to be over-expressed in different cancers including lung cancer^15,23,39,40^ and their high expression was correlated with poor overall survival^23^. NSCLC tissues expressing B7-H3 or B7-H4 had fewer T infiltrating lymphoid cells (TILs) and significantly more common lymph node metastasis^41^. B7-H5 is a ligand for CD28H and acts as a co-inhibitory molecule. The expression of B7-H5 is predominantly found in hematopoietic tissues, Treg cells and myeloid cells^15,42^. B7-H5 is also widely expressed in tumor cells and high expression of it is related to a worse prognosis in various cancers including lung cancer^24^. It is well known that EGFR is notorious for being widely expressed and mutated in NSCLC^43^. It participates in immune evasion, most likely by regulating the expression of B7-H5^44^. B7-H6 is a ligand of NK cell activating receptor NKp30. B7-H6 can be induced by inflammatory stress in healthy cells, and is also expressed on various primary human tumors^15,45^. Although abnormal over-expression of B7-H6 was linked with poor prognosis in various types of cancers^46,47^ its expression in NSCLC is limited^48^.

Promoter DNA methylation, as an important epigenetic modification, has a widespread association with gene expression^49,50^. Therefore, we further investigated whether promoter DNA methylation were involved in B7 member dysregulation. As shown in Fig. 1c, the expression of most B7 members were significantly and negatively correlated with promoter DNA methylation in LUAD while only B7-H1 and B7-H4 were significantly associated with DNA methylation in LUSC. On the other hand, the expression of almost all B7 members was significantly associated with copy number alterations (Fig. 1d). These results suggested that both DNA methylation and copy number alterations might be involved in the altered expression of B7 members in NSCLC. Similar with our results, promoter methylation has also been reported to show regulatory effect on the expression of B7-1/2/H3/H4/H5/H6/H7 in previous studies^51–53^. Since the expression of B7 members was significantly related to copy number alterations, we then further determined the mutation frequency and mutation rate of B7 family members in NSCLC (Fig. 2a, b), including mutation, amplification and deep deletion. The overall mutation frequency and mutation rate of B7 family in LUSC were higher than that of LUAD. The mutation rates were relatively higher for B7-1and B7-2 in LUSC and also for B7-DC, B7-H1 and B7-H4 in both subtypes. Amplification accounted for the majority of mutation in B7 members with increased expression, such as B7-1, B7-2, B7-H3, B7-H5, B7-H6 in both NSCLC subtypes and B7-H2 and B7-H7 in LUSC. Deep deletion was frequently found in B7-DC, B7-H1 and B7-H4 in LUAD and LUSC. Consistent with our results, gene amplification of B7-H1 (PD-L1) has been demonstrated by several studies^54–56^ and was associated with response to nivolumab monotherapy among patients with NSCLC^56^. Shallow or deep deletion of B7-H1 was reported in head and neck cancer^57^. Deep deletion was also a major mutation type for B7-DC and B7-H1 in gastric cancer^53^ and breast cancer^47^ and for B7-H4 in signet ring cell carcinoma of the stomach^53^.

To find the potential targets and downstream pathways of B7 family, differentially expressed proteins influenced by B7 family were identified and subjected to KEGG and GO enrichment analysis (Fig. 4a–c). Intriguingly, these differential protein-coding genes were commonly enriched within ErbB signaling pathway and three GO processes mainly involving protein binding, cytosol and signal transduction in both NSCLC subtypes. ErbB signaling pathway is frequently overly activated in malignancies of epithelial origin and signaling from ErbB receptors engaged in every cellular process including proliferation, survival, migration and differentiation and cell and tissue morphogenesis^58^. Epithelial growth factor receptor (EGFR also ERBB1or HER1) has been the most well studied of the four members of the ErbB family. Over 60% of NSCLC patients express EGFR and tyrosine kinase inhibitors (TKIs) targeting the kinase domain of EGFR are clinically effective in NSCLC patients harboring activating mutations in the tyrosine kinase domain of the EGFR gene^43^. The role of EGFR in regulating PD-L1 expression in NSCLC has been reported by several studies^59–61^. One study found that activation of EGFR and mutations of 19 deletion or L858R induced PD-L1 expression via the p-ERK1/2/p-c-Jun signaling pathway^61^. The other study revealed that EGFR up-regulated PD-L1 expression through the IL-6/JAK/STAT3 signaling pathway^62^. Both studies found that treatment with EGFR TKIs reduced the expression of PD-L1. However, the clinical efficacy of anti-PD-1/PD-L1 immune checkpoint inhibitors (ICIs) in advanced EGFR mutant NSCLC or combination therapy of EGFR-TKIs and anti-PD-1/PD-L1 ICIs is still not clear yet^63^. Research evidence and clinical trials showed that combination of anti-PD-1/PD-L1 ICIs and EGFR TKIs did not exhibit synergistic anticancer effect in NSCLC^61,63^. It is suggested that anti-PD-1/PD-L1 ICIs in combination with chemotherapy or other targeted therapy might be more effective in treating advanced NSCLC^63^. In addition to EGFR, research evidence showed that *ERBB2* and *ERBB3* mutation could also upregulate PD-L1 expression and suppress normal T-cell-mediated cytotoxicity through activation of the PI3K/Akt signaling pathway^64^. Together, our findings stress a close relation between ErbB signaling and B7 family expression, which is partially supported by the literature and awaits further investigation.

At the same time, our results demonstrated that MAP2K1 (MEK), MAPK1 (ERK, also ERK2) and MAPK3 (ERK1) were important B7 family related genes in both NSCLC subtypes and were positively correlated with several B7 family members (Fig. 5). The activation of MEK/ERK pathway has been reported for different B7 members. According to the literature, B7-H4 contribute to cancer progression by activation of the ERK 1/2 signaling^65,66^. B6-H6 could also promote non-hodgkin lymphoma via Ras/MEK/ERK pathway^67^. B7-H3 regulates breast cancer stemness by activating MEK/MAPK signaling^68^. In colorectal cancer, PD-L1 upregulates HMGA1 to activate PI3K/Akt and MEK/ERK pathways to promote cancer stem cell expansion^69^. On the other hand, the ERK-MAPK pathway has been shown to regulate PD-L1 expression in different cancer types^70–72^. ERK and JNK pathways are also involved in IFN-γ-induced B7-DC expression on tumor cells^73^. Interestingly, out findings revealed a strong positive correlation between MEK/ERK and B7-H3 and B7-H6 in NSCLC which has been rarely studied. One study showed that combination therapy of MEK inhibitor with B7-H3-redirected bispecific antibody significantly suppressed in vivo tumor growth and increased T cell infiltration^74^. Synergistic antitumor effect has also been observed for MEK inhibitors combing with anti-PD-1 or anti-PD-L1 antibodies in different *ex-vivo* and in vivo models in various cancers^75–78^. The use of compounds targeting Ras-Raf-MEK-ERK signaling pathway, such as RAF or MEK inhibitors, has led to substantial improvement in clinical outcome in various types of tumor^75^. However, resistance to these agents is usually found. Several orally effective, potent, and specific inhibitors of ERK1/2 are in early clinical trials^79^. These results underline the potential of combination treatment combining MEK or ERK inhibitors with PD-1/PD-L1/B7-H3/B7-H6 blockade.

To date, the epidermal growth factor receptor (EGFR/ERBB1) distinctive features of the ERBB family provides a foundation for the design of therapies targeting NSCLC^58,80^. Tyrosine kinase inhibitors (TKIs) treatment has been constantly updated and combined with other treatment options to improve prognosis of NSCLC^80,81^. The Shc-activated or Grb2-activated mitogen-activated protein kinase (MAPK) pathway is a common downstream signaling of ErbB receptors (Fig. S1c). MAP2K1 (MEK), MAPK1 (ERK) and MAPK3 (ERK1)play a pivotal role in regulating cell proliferation, transcriptional regulation, differentiation and survival as members of the MAPK signaling pathway^82^. Collectively, our study supports a close link between B7 family and the ErbB/MAPK signaling pathway in NSCLC. More in-depth exploration of TKIs or MEK/ERK inhibitors combining with B7 checkpoint inhibitors will be help for designing rational immunotherapy for NSCLC.

## Methods

### Data analysis

Data were extracted from The Cancer Genome Atlas (TCGA) (http://cancergenome.nih.gov/) and Genotype-Tissue Expression (GTEx) (https://www.gtexportal.org/home/), expression levels of B7 family members were log2-transformed and matched clinical data in NSCLC.GISTIC 2.0 database and reversedphase protein array(RPPA) from cBioportal website (https://www.cbioportal.org/) were used for data visualization, such as somatic mutations, copy number variations, DNA methylation and screening of differentially expressed proteins^83^. The relationship between mRNA expression levels of B7 family members and DNA methylation or copy number changes was analyzed using cBioportal database.

### Differential analysis

Differential analysis of patients with LUAD and LUSC according to whether each B7 family member is mutated on the cBioportal website. *P* value < 0.05 is identified as differentially expressed proteins. All information of differential analysis corresponding to each B7 family member is deposited in Supplementary table 1 and 2.

### Protein and functional network analysis

The PPI network is constructed using STRING and Cytoscape software. The STRING online database (https://string-db.org/) has powerful visualization and customization capabilities to annotate protein–protein interactions (PPI) network^84^. CytoHubba plugin of Cytoscape provides 11 topological analysis methods to identify important nodes in biological networks^85^.

### GO and KEGG enrichment analysis

The screened differentially expressed proteins were subjected to Gene Ontology (GO) and Kyoto Encyclopedia of Genes and Genomes (KEGG) analysis in the DAVID function annotation tool (https://david.ncifcrf.gov). DAVID is a database of biological information that integrates biological data and analysis tools to provide systematic and comprehensive biological functional annotation information for large-scale gene or protein lists (hundreds of genes or protein ID lists)^86,87^. The abundance of biological processes (BP), molecular functions(MF), and cellular components(CC) was analyzed, and the influenced metabolic processes were obtained by consideration of gene counts and *P* value.

### R project analysis

Kaplan–Meier analysis was performed using survminer R package. Comparison of functional similarity of BP, MF and CC between proteins was constructed using GOSemSim and org.hs.eg.db packages. The R package of GOSemSim depended on GO annotation information to compute the semantic similarity among GO terms, gene products, and gene clusters^88^. From this analysis, major genes contributed to GO analysis can be found. Corrplot package was used to characterize the extent of co-expression between differentially expressed genes by computing gene expression. The ggpubr package was utilized to display mRNA expression levels in normal and tumor tissues.

### Statistical analysis

GraphPad Prism 7 (GraphPad Software, La Jolla, CA) was used for statistical analysis. Student's t-test was utilized to compare differences between two groups. (*P < 0.05; **P < 0.01 and ***P < 0.001) One-way ANOVA was utilized to compare multiple groups. Spearman's and Pearson's correlation analysis was utilized to calculate the correlation between the two groups. Kaplan–Meier analysis was used to analyze the overall survival rate based on TCGA data, and the *P* value was calculated by log-rank tests. *P* < 0.05 was considered as statistically significant.

## Supplementary Information

Supplementary Figure 1.

Supplementary Table 1.

Supplementary Table 2.

## Data Availability

The datasets used and/or analyzed during the current study are available from the corresponding author on reasonable request.
