# Supplementary material for: Comprehensive characterization of B7 family members in NSCLC and identification of its regulatory network
Source: Sci Rep. 2023 Mar 15;13:4311. doi: 10.1038/s41598-022-26776-w (PMC10017798; doi:10.1038/s41598-022-26776-w)
Supplement: Supplementary file 1 — Supplementary Figure 1. [file 41598_2022_26776_MOESM1_ESM.docx]

**
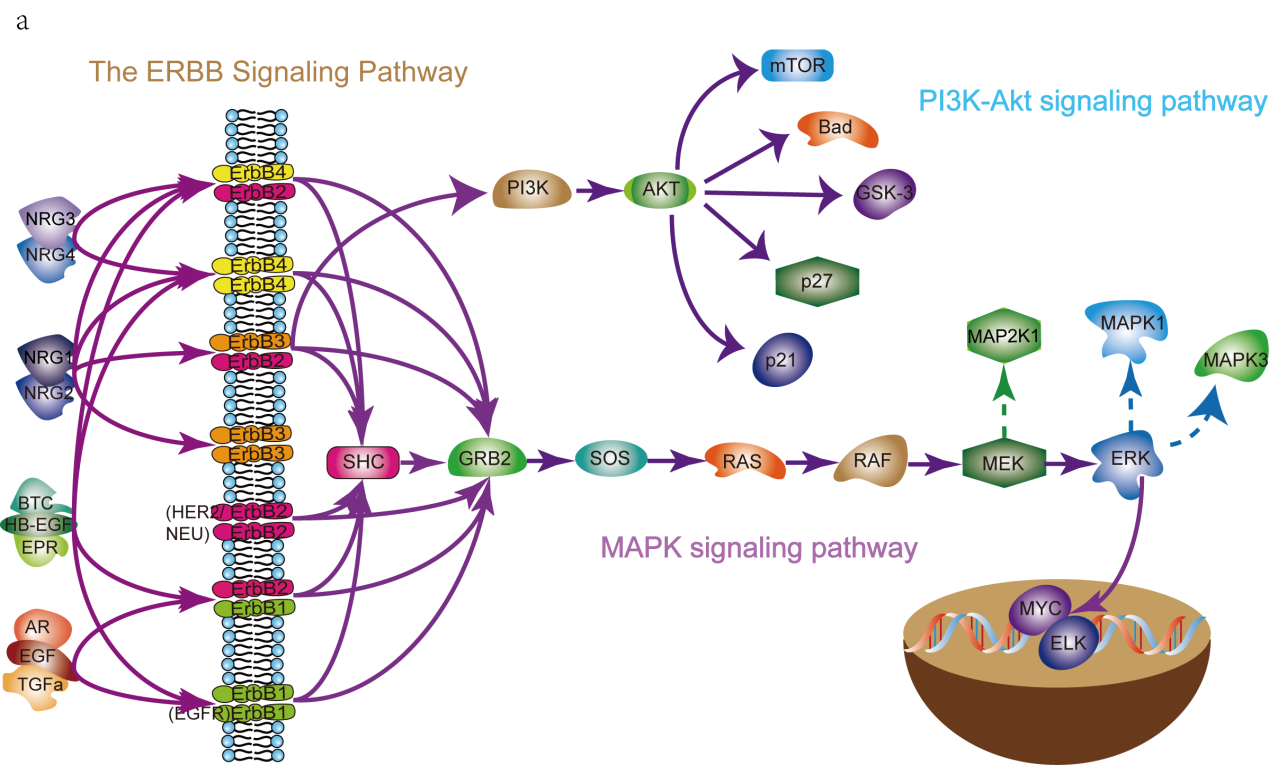
**

**Supplementary Fig 1.signaling pathway of Critical differentially expressed proteins**

(a) ErbB/MAPK signaling pathway and ErbB/PI3K-Akt signaling pathway are pivotal pathways of B7 family in regulating NSCLC.
